# Evaluating the Usefulness of Human DNA Quantification to Predict DNA Profiling Success of Historical Bone Samples

**DOI:** 10.3390/genes14050994

**Published:** 2023-04-27

**Authors:** Jacqueline Tyler Thomas, Courtney Cavagnino, Katelyn Kjelland, Elise Anderson, Kimberly Sturk-Andreaggi, Jennifer Daniels-Higginbotham, Christina Amory, Brian Spatola, Kimberlee Moran, Walther Parson, Charla Marshall

**Affiliations:** 1 Armed Forces Medical Examiner System’s Armed Forces DNA Identification Laboratory (AFMES-AFDIL), Dover Air Force Base, Dover, DE 19902, USA; 2 SNA International, LLC (Contractor Supporting the AFMES-AFDIL), Alexandria, VA 22314, USA; 3 Amentum Services Inc. (Contractor Supporting the AFMES-AFDIL), Germantown, MD 20876, USA; 4 Institute of Legal Medicine, Medical University of Innsbruck, 6020 Innsbruck, Austria; 5 National Museum of Health and Medicine, Anatomical Division, Defense Health Agency, Silver Spring, MD 20910, USA; 6 Forensic Science Program, Department of Chemistry, Rutgers University-Camden, Camden, NJ 08102, USA; 7 Forensic Science Program, The Pennsylvania State University, University Park, State College, PA 16802, USA

**Keywords:** qPCR, ancient DNA, forensic DNA, massively parallel sequencing, next generation sequencing, SNP, mtDNA, STR typing

## Abstract

This study assessed the usefulness of DNA quantification to predict the success of historical samples when analyzing SNPs, mtDNA, and STR targets. Thirty burials from six historical contexts were utilized, ranging in age from 80 to 800 years postmortem. Samples underwent library preparation and hybridization capture with two bait panels (FORCE and mitogenome), and STR typing (autosomal STR and Y-STR). All 30 samples generated small (~80 bp) autosomal DNA target qPCR results, despite mean mappable fragments ranging from 55–125 bp. The qPCR results were positively correlated with DNA profiling success. Samples with human DNA inputs as low as 100 pg resulted in ≥80% FORCE SNPs at 10X coverage. All 30 samples resulted in mitogenome coverage ≥100X despite low human DNA input (as low as 1 pg). With PowerPlex Fusion, ≥30 pg human DNA input resulted in >40% of auSTR loci. At least 59% of Y-STR loci were recovered with Y-target qPCR-based inputs of ≥24 pg. The results also indicate that human DNA quantity is a better predictor of success than the ratio of human to exogenous DNA. Accurate quantification with qPCR is feasible for historical bone samples, allowing for the screening of extracts to predict the success of DNA profiling.

## 1. Introduction

DNA profiling of historical remains can be notoriously difficult. As DNA degrades over time, only small fragments can be extracted from aged skeletal samples. Over the past 10 years, ancient DNA (aDNA) extraction methods have been developed and optimized to recover ultrashort (<40 bp) DNA molecules [1,2]. These small DNA fragments are more conducive to shotgun library preparation and next-generation sequencing (NGS) than PCR-based DNA profiling methods that require intact amplicon targets. In addition to fragmentation, DNA damage such as cytosine deamination often affects ancient and historical remains (e.g., [3]). This can result in base substitutions in the replication process, which can impact the interpretation of sequence data. Poor environmental conditions, such as a warm and humid climate, can exacerbate DNA degradation and lead to microbial growth. Human DNA can be overwhelmed by co-extracted exogenous DNA, including DNA from the soil [4], which may be more abundant than the endogenous DNA in the specimen. The microenvironment and the skeletal element may also play a role in the human DNA content [5]. Finally, human remains are sometimes stored in conditions that are not conducive to DNA preservation, such as in plastic bags [6], or they may be treated with chemical preservatives such as formalin [3] that further diminish the ability to recover DNA.

Due to these myriad factors, it can be difficult to gauge sample success prior to devoting resources to sample extraction, library preparation, and sequencing, as noted in Kapp et al. [7]. In some situations, there may be an absence of contextual or historical information that could help determine the degree or types of damage in a sample prior to lab processing. Screening methods, such as whole genome sequencing (WGS), are often utilized in the aDNA community to predict sample success. However, this method is costly and minimally effective for samples with <1% endogenous DNA [8]. Various aDNA laboratories have implemented hybridization capture for these compromised samples to target the endogenous portion. Some laboratories have implemented custom quantitative real-time PCR (qPCR) assays for a variety of species to quantify endogenous DNA in sample extracts and prepared libraries with the intent of predicting downstream success. Similarly, Enk et al. [9] found that multi-copy qPCR assays can successfully estimate target DNA fragment length, which may improve the predictive power of qPCR as a screening tool. This conclusion was supported by a comparison of quantification methods conducted by Brzobohatá et al. [10] using skeletal remains from a medieval burial site. Total double-stranded DNA (dsDNA) quantification methods resulted in the highest DNA concentrations due to the presence of exogenous DNA, while qPCR underestimated DNA quantity in degraded samples with small fragment sizes. A quantification system that utilized capillary electrophoresis to determine DNA concentration and fragment distribution was the most accurate and informative method of aDNA quantification for the data presented in [10].

Within the forensic community, sample screening is traditionally performed with test PCR amplification events or qPCR. The most utilized human qPCR assays have been developed to work in tandem with short tandem repeat (STR) typing kits, such that qPCR results can identify the amount of target DNA of suitable size to amplify successfully with an STR multiplex. The forensic qPCR assays are furthermore designed with an internal positive control (IPC) that can alert the user to the presence of inhibitors common in forensic samples. Current qPCR kits include multi-copy autosomal and Y-chromosomal DNA targets, and often two autosomal targets of different fragment lengths are used for a degradation assessment (degradation index). The suitability of these traditional nuclear qPCR assays for highly degraded DNA has yet to be shown. As an alternative to nuclear DNA quantification, the use of qPCR to quantify mitochondrial DNA (mtDNA), which is more readily recovered from historical samples, has proven successful. MtDNA qPCR has been used to assess fragmentation and to direct the processing of degraded remains in both ancient [11] and forensic [12] settings.

This proof-of-concept study evaluated DNA quantification as a screening method for historical remains when processed through a variety of downstream DNA profiling modalities including single nucleotide polymorphism (SNP), mtDNA, autosomal STR (auSTR), and Y-STR genotyping. A genomic DNA quantification method with an intercalating dye was used to determine the total dsDNA and well-characterized qPCR kits were used to determine human DNA quantity. The qPCR kits assessed included PowerQuant, Quantifiler Trio (Trio), and Investigator Quantiplex Pro (Pro). These assays are human-specific and unaffected by overwhelming quantities of non-human DNA. Together, this quantification data (total and human) can be used to calculate a human DNA (hDNA) ratio that also accounts for the proportion of exogenous DNA in a sample. This study tested whether a range of aged and highly degraded samples contained sufficient DNA to be detectable with three forensically oriented commercial qPCR kits. It then compared both the human DNA quantity and the human DNA ratio as potential screening tools for forensic DNA profiling to determine the most beneficial processing workflow. Such a predictive tool could increase efficiency and better direct resources, even in scenarios where little to no contextual sample information is available.

## 2. Materials and Methods

### 2.1. Skeletal Samples

Thirty-one burials, from six historical contexts, were included in this study for DNA analysis (Table 1). These samples range in age from approximately 80 to 800 years postmortem and originated from multiple locations across the United States and Europe. The JB55 [13], Arch Street, and World War II (WWII) samples were previously processed at the Armed Forces Medical Examiner System’s Armed Forces DNA Identification Laboratory (AFMES-AFDIL), ensuring that a range in sample quality was assessed prior to inclusion in the study. The burial of JB55 also referred to as the New England vampire, was excavated in 1990 after being discovered during sand and gravel operations [13]. In addition to JB55, a second bone known to be from a red deer through prior mtDNA testing, was included to test the specificity of the qPCR assay. Non-human bones may be submitted for forensic DNA analysis when they lack diagnostic features for definitive anthropological species identification. The Arch Street project consisted of remains from the First Baptist Church of Philadelphia that were unearthed during construction in 2017. WWII samples were comprised of disinterred remains from Germany and the National Memorial Cemetery of the Pacific, also known as the Punchbowl. The WWII Punchbowl samples were likely impacted by chemical treatments, such as formalin embalming and the application of hardening compounds, that inhibit DNA testing [14]. However, the exact chemical treatment of each Punchbowl burial tested here was unknown, as these are true historical cases with only generalized contextual information [3]. The Harewood Cemetery is located on the family estate of Samuel Washington, brother of George Washington who served as the first president of the United States. Of the three burials included in this study, a single skeletal element was selected per grave, aside from one burial which had two elements tested. The skeletal remains associated with the Basilica of St. Mary’s Assumption of the former monastery church in Fürstenfeldbruck, Germany, were collected from a walled-up, uninscribed coffin compartment in the crypt. The remains were collected in the presence of HRH Duke Franz of Bavaria and were radiocarbon dated to the mid-13th to early 14th centuries. Overall, samples exhibited varying levels of degradation due to environmental factors and chemical treatment. Additional information about each sample can be found in Table S1.

### 2.2. Sample Preparation and DNA Extraction

All DNA extractions were performed at the AFMES-AFDIL, with the exception of the 12 Basilica samples that were extracted at the Institute of Legal Medicine in Innsbruck. DNA extractions were performed according to ancient [15] and forensic [16] DNA standards. This included the use of dedicated low-copy extraction laboratories with positive pressure for extraction and library preparation prior to amplification, appropriate personal protective equipment, sterilization of tools and laboratory consumables, and inclusion of extraction and library preparation controls processed alongside samples.

For the extractions performed at the AFMES-AFDIL, the outer surface of the bone samples and excess spongy bone were sanded to remove exogenous DNA. Bone fragments were washed with sterile water and 100% ethanol, then ground to a powder in a Waring blender cup (Waring, Torrington, CT). Arch Street samples underwent a bleach wash with a 70 mM solution of household bleach (8.25% sodium hypochlorite) prior to the sterile water and ethanol washes. Bone powder aliquots and associated reagent blanks (RB) underwent the Dabney extraction protocol described in Rohland et al. [2] to retain ultrashort DNA fragments. The volume of Dabney extraction buffer (0.46 M EDTA, 0.05% Tween-20) and proteinase K (20 mg/mL) differed across sample sets depending on whether the original AFDIL Dabney protocol or the modified AFDIL Dabney protocol was used (Table S1). The AFDIL Dabney protocol utilized 0.2 g of bone powder, 1 mL of Dabney extraction buffer, and 25 µL of proteinase K. The modified protocol utilized up to 0.4 g bone powder, 4 mL Dabney buffer, and 200 µL of proteinase K to enhance digestion. Both the AFDIL Dabney and the modified AFDIL Dabney methods maintained the same 10X binding buffer to lysate ratio as utilized in the Rohland et al. [2] Dabney method for small fragment retention. All samples, across both protocols, were incubated at 56 °C overnight with constant agitation. Following overnight incubation, the supernatant was separated from the remaining bone powder and combined with 10X QIAGEN PB buffer (QIAGEN, Hilden, Germany) in place of binding buffer ‘D’ used in the Rohland et al. method [2]. Samples were spun through a Roche High Pure Viral silica-based column (Roche, Pleasanton, CA, USA), and the bound DNA was washed twice with QIAGEN PE buffer. DNA was eluted in 50–60 µL Tris-EDTA (10 mM Tris, 0.1 mM EDTA, pH 7.5) following the Rohland et al. protocol [2]. Extracts were stored at −20 °C immediately upon completion until downstream processing was initiated (approximately one to six months). Replicate extracts were prepared and homogenized to provide sufficient volume for quantification, STR typing and NGS analysis.

The bone samples processed at the Institute of Legal Medicine in Innsbruck were sanded using a Dremel tool until 1–2 mm of the surface layer was removed. The bone powder was retrieved using a dental drill at low speed to avoid overheating the tissue. The resulting powder was lysed in three parallel 50 mg aliquots, and DNA was extracted by combining the aliquots through a filter, following the protocol described by Xavier et al. [17]. Non-template controls and RBs were run in parallel throughout the experimental procedure to monitor possible contamination. Upon receipt at the AFMES-AFDIL, sample extracts ranged in volume from 13–34 µL and 10 mM Tris-HCl (pH 8.5) was added to reach a final volume of 45 µL prior to downstream processing.

### 2.3. DNA Quantification

Human nuclear DNA-specific qPCR was performed using an Applied Biosystems 7500 Real-Time PCR System (Thermo Fisher Scientific, Waltham, MA, USA). For comparison purposes, DNA was quantified with three qPCR kits, unless indicated (Table S1). The Investigator Quantiplex Pro kit (QIAGEN), Quantifiler Trio kit (Thermo Fisher Scientific), and PowerQuant System (Promega Corporation, Madison, WI, USA) were used following their respective manufacturer’s recommendations. These three kits include both a small and large human autosomal target and a small Y-chromosomal target, all multi-copy. The PowerQuant System also includes a large Y-chromosomal target to assess Y chromosome-specific degradation. DNA was quantified using 2 µL extract. All kits include an IPC, a synthetic target that is used to detect the presence of some common inhibitors. A non-human bone sample was also tested in conjunction with standards and non-template controls. The size of each target can be found in Table 2. Where possible, the presentation of qPCR and sample processing information complies with the Minimum Information for Publication of Quantitative Real-Time PCR Experiments guidelines [18]. Results from the small autosomal target of each kit were compared in a pairwise fashion using a two-tailed, paired *t*-test. An α value of 0.05, consistent with a 95% confidence interval, was selected for assessing statistical significance. A *p*-value > 0.05 was considered ‘not significant’.

The Basilica extracts were also quantified with SD quants, a tetraplex qPCR system, targeting an IPC, one nuclear (70 bp), and two mtDNA (69 bp and 143 bp) targets according to Xavier et al. [11]. The mtDNA results were given in mitogenome equivalents (mtGE) while pg/µL were used for the autosomal target. A degradation index was calculated based on concentrations of the mtDNA targets.

Total dsDNA was quantified using the Qubit 2.0 Fluorometer and the Qubit dsDNA HS or BR Assay kit according to the manufacturer’s instructions (Thermo Fisher Scientific) using 2 µL of extract.

To assess whether the proportion of human-to-exogenous DNA was predictive of downstream success, an hDNA ratio was determined using the formula below for each sample. The calculation required the quantification results from a target-specific qPCR assay (small autosomal target) and the total dsDNA quantity. The ratio compared the picograms of human autosomal DNA (auDNA) to nanograms of total dsDNA to make the resulting ratios manageable numbers.

$$\mathrm{hDNA}\;\mathrm{Ratio}=\left(\frac{\mathrm{pg}\;\mathrm{input}\;\mathrm{based}\;\mathrm{on}\;\mathrm{qPCR}\;\mathrm{small}\;\mathrm{autosomal}\;\mathrm{target}}{\mathrm{ng}\;\mathrm{input}\;\mathrm{of}\;\mathrm{total}\;\mathrm{dsDNA}}\right)$$

### 2.4. Next Generation Sequencing

#### 2.4.1. Library Preparation

Sample and control libraries were prepared at the AFMES-AFDIL in a dedicated low copy, pre-PCR laboratory using the KAPA HyperPrep Kit (Roche Sequencing, Wilmington, MA, USA) following the procedure described in Tillmar et al. [19] unless noted. No DNA repair was performed on the extracts. DNA input for end repair was based on the Qubit values. Adapter ligation was performed using 15 µM KAPA unique dual-indexed adapters (Roche Sequencing) for all samples and RBs. A 1:10 adapter dilution was used for all negative and positive controls based on a DNA input ≤ 1 ng. A 1.3x AMPure XP (Beckman Coulter, Brea, CA, USA) ratio was used for purification following adapter ligation to ensure the retention of small DNA fragments. Library PCR was performed using KAPA HiFi HotStart Uracil+ Ready Mix and 20 µM Illumina Primer mix (Roche Sequencing). The Uracil+ Ready Mix contains a uracil-tolerant polymerase that allows for the amplification of DNA with cytosine deamination, as characteristic of ancient and historical DNA [3,20]. After PCR amplification, a 5X QIAGEN MinElute (QIAGEN) purification was performed with elution in 25 µL Tris-EDTA. Library quality was evaluated using the 2100 Bioanalyzer instrument (Agilent Technologies, Santa Clara, CA, USA) before proceeding with hybridization capture.

Additionally, a library input reduction test was performed to assess whether using the maximum 1 µg dsDNA input for library preparation resulted in inhibition and thus poor-quality libraries. Three samples from different historical contexts with initial library inputs of a maximum of 1 µg were diluted to 500 ng and 200 ng to evaluate reduced library input. Library preparation was performed on the 500 ng and 200 ng samples using the same procedures described previously.

#### 2.4.2. Hybridization Capture

Purified libraries were enriched via hybridization capture using myBaits v5 kits (Arbor Biosciences, Ann Arbor, MI, USA). Sample libraries were captured with two bait sets unless indicated (Table S1). The forensically oriented FORCE SNP panel [19] β version contains ~20,000 unique baits targeting 5402 SNPs including identity, ancestry, phenotype, X- and Y-chromosomal SNPs. The β version excludes 44 tri-allelic, clinically relevant, uninformative and/or underperforming SNPs identified in [19]. The second bait set was a custom panel that covers the human mitogenome with 1800 unique oligos [14]. A maximum of 7 µL of the library was used for each capture reaction (FORCE and mitogenome). The capture product was amplified and purified according to [19], except the 20 µM Illumina Primer mix was used. Samples were eluted in 20 µL Tris-EDTA.

All captured libraries were run on a 2100 Bioanalyzer using the 7500 assay. Any libraries with total DNA concentrations over 50 ng/µL were diluted and re-run using the 7500 assay for more accurate quantification.

#### 2.4.3. Normalization and Pooling

FORCE and mitogenome hybridization capture samples were primarily pooled by normalization, with a 50 nM target for the 170–1000 bp range. The mitogenome capture samples for the Basilica and Arch Street were pooled by volume instead of normalizing. Mitogenome sequencing pools contained 21–27 samples, while FORCE pools consisted of 11–16 samples. Positive controls were not sequenced and were used only to monitor library preparation and capture success.

#### 2.4.4. Loading and Sequencing

All pools were quantified on the 2100 Bioanalyzer using the DNA 7500 kit and diluted to 4 nM prior to loading. The 4 nM pools were denatured and diluted to 1.0 pM with 2.5% Illumina PhiX Control V3 (PhiX) (Illumina) or 1.2 pM with 5% PhiX. All runs were performed on a NextSeq 550 (Illumina) with Mid output v 2.5 kits. As general sample qualities were known, pools containing only highly degraded samples were sequenced with 75 × 75 paired-end cycles. For samples with moderately degraded DNA, 150 × 150 paired-end sequencing was used.

#### 2.4.5. Sequence Data Analysis

Following demultiplexing, FASTQ files were imported into the CLC Genomics Workbench v12.0.1 (QIAGEN) for analysis. FORCE analysis was completed with a custom workflow as described in [19] with two modifications: (1) reads less than 30 bp were discarded to minimize the mapping of non-specific reads and (2) a custom de-duplication tool that mimics the functionality of markdup from SAMTools v1.61.1 (http://www.htslib.org/doc/samtools-markdup.html) (accessed on 14 February 2023) was used to more effectively remove PCR duplicates. Genotypes were called using the Known Mutations from Mapping tool, and a custom Microsoft Excel (Redmond, WA, USA) template as described in [19] was used to apply additional analysis parameters to called SNPs and simplify the output. Calling of target SNPs required a minimum read count (coverage) of 10X. In addition to the minimum coverage requirement, an allele frequency of 90% or greater was necessary for homozygous genotypes. Heterozygous genotypes needed a minor allele frequency (MAF) of at least 30% to be called. Imbalanced genotypes (10–30% MAF) were excluded from analysis (not called), as heterozygosity could not be confidently determined. SNP coverage at 1X and 5X was also evaluated.

Mitogenome analysis was also completed in the CLC Genomics Workbench v12.0.1 with the AFDIL-QIAGEN mtDNA Expert (AQME) plug-in and a custom workflow, as described in [14,21]. Reads were mapped to the revised Cambridge Reference Sequence (rCRS) [22,23] using the same stringent mapping parameters incorporated into the custom FORCE workflow. The custom de-duplication tool with local realignment was implemented to remove PCR duplicates and assist in indel alignment. A 10X coverage threshold, with a variant frequency ≥10% and a minimum variant count of four, was required for variant calling.

### 2.5. STR Typing

To assess the suitability of DNA quantification to predict PCR enrichment success, short tandem repeat (STR) typing was performed. The PowerPlex Fusion kit (Promega) (PPF) amplifies 22 forensic auSTRs, one Y-STR, and one non-STR sex-determination locus (amelogenin). Samples, along with a 2800M positive control and negative control (sterile water), were amplified according to the manufacturer’s recommendations. The maximum sample volume of 15 µL was used when the extract volume allowed, unless indicated (Table S1).

The Applied Biosystems’ AmpFLSTR Yfiler kit amplifies 17 forensic Y-chromosomal STRs (Y-STRs) for male identification. A modified Yfiler approach optimized for degraded, low copy number samples (LCNY) was developed at the AFMES-AFDIL [24]. This LCNY protocol, which includes increased polymerase and cycles, was used to generate all Y-STR data. The maximum sample volume of 9.2 µL was utilized when extract the volume allowed, unless otherwise indicated (Table S1). If inhibition was suspected, a range of input volumes was amplified in a repeat amplification event to improve the number of loci obtained.

The amplified product was prepared for capillary electrophoresis according to the manufacturer’s manual for each kit and loaded on a 3500xL Genetic Analyzer with POP-4 polymer (Applied Biosystems, Foster City, CA, USA). Samples were injected for 15 s (PPF) or 7 s (LCNY) using default injection parameters for each kit.

STR data were analyzed using GeneMapper ID-X v1.4 (Applied Biosystems). Locus-specific stutter filters were determined by internal validation at the AFMES-AFDIL. For PPF, the reporting threshold was 70 relative fluorescent units (RFU) for heterozygous alleles and the Y-STR locus, and 550 RFU for homozygous alleles at the auSTR loci. For LCNY, peaks over 70 RFU were considered reportable.

## 3. Results

### 3.1. DNA Quantification

**Figure 1:**
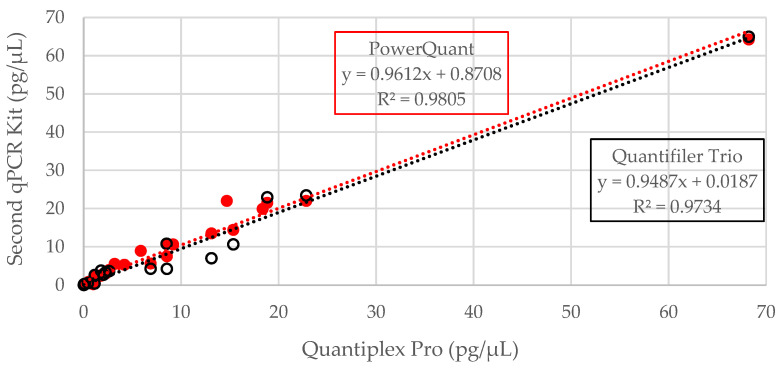
Small autosomal target results for Quantiplex Pro (91 bp) compared to Quantifiler Trio (80 bp) (black outline, 19 samples) and PowerQuant (84 bp) (solid red, 31 samples). Equations for each trendline are outlined in the same color as the associated line and data points.

All 32 samples were quantified with Pro and PowerQuant; a total of 20 samples were quantified with Trio (Table S2). All human samples produced amplifiable DNA using the small autosomal target of each kit tested, except for one sample with PowerQuant and a different sample with Trio. These two samples were both from the WWII Punchbowl context and likely underwent chemical treatment known to cause inhibitory DNA-protein crosslinks [3,14]. All human samples produced amplifiable DNA with the small target of Pro. At least one-third of samples did not produce amplifiable DNA (undetermined result) for the large autosomal target of each kit; therefore, the degradation index was incalculable. As a result, the small autosomal target was the focus of the comparisons and analyses presented in this study. A 1:1 relationship was observed between kits when DNA concentrations were compared in a pairwise fashion (Pro vs. PowerQuant, Pro vs. Trio, PowerQuant vs. Trio) (Figure 1 and Figure S1), with R^2^ values greater than 97%. Thus, the DNA concentrations obtained from the three qPCR kits were concordant. Nine of the 11 extracts with values less than 5 pg/µL with Pro resulted in significantly higher values with Trio (*p* = 0.02; two-tailed, paired *t*-test). These nine samples were known to be highly degraded, and thus the smaller target size (80 bp for Trio compared to 91 bp for Pro) could explain this discrepancy. The PowerQuant system resulted in slightly higher quantification results for 71% of samples compared to Pro, perhaps due to the smaller target for PowerQuant relative to Pro; however, the difference was not significant (*p* = 0.11; two-tailed, paired *t*-test). Quantification results from the small autosomal targets were compared to the approximate years postmortem to determine if age alone was predictive of DNA extract concentration. No direct correlation was observed (Figure S2). As quantification results across the three kits were consistent, results from downstream processing will be presented primarily in reference to Pro since more bone sample data points were available for this kit.

**Figure 2:**
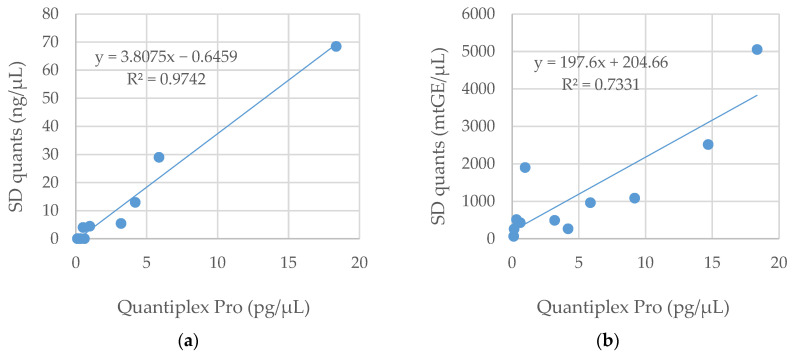
Comparison of Quantiplex Pro small autosomal target (91 bp) and SD quants (**a**) small autosomal target (70 bp) and (**b**) small mtDNA target (69 bp) across 12 Basilica samples. mtGE—mitochondrial genome equivalent.

In addition to the three qPCR systems tested at the AFMES-AFDIL, one set of samples (Basilica) was quantified with the SD quants tetraplex at the Medical University of Innsbruck. Two extracts resulted in no quantification value with the SD quants system but did return a concentration with Pro (Figure S2). Excluding these two outliers, the R^2^ value improved from 0.435 to 0.974 (Figure S3 and Figure 2a). The DNA concentrations produced from the autosomal target of the SD quants tetraplex were approximately four times higher than Pro (Figure 2). This may be due in part to the smaller target of the SD quants system (70 bp for SD quant and 91 bp for Pro), resulting in more amplifiable DNA from these degraded sample extracts.

The mtDNA portion of the SD quants qPCR assay included both a small (69 bp) and a large (143 bp) target; however, only the small target was considered here due to the degraded nature of the ancient Basilica samples tested. While the R^2^ between the small mtDNA target and Pro was only moderate (73%), the trend indicates approximately 1000 mtGE for each 5 pg/µL human autosomal DNA (Figure 2b). This ratio of mtDNA to nuclear DNA seems plausible and has been previously observed in a different context, (e.g., [15]).

Quantification results for the small Y-DNA targets were compared across the three qPCR kits in a similar manner as the small autosomal targets. Trio and PowerQuant were both compared directly to Pro and to one another (Figures S4 and S5), resulting in R^2^ values of 0.95 and 0.92, respectively. Trio and PowerQuant both produced undetermined results for samples that generated a positive Y-DNA quantification result in Pro (Table S2).

All RBs and the non-human bone sample failed to result in any amplifiable human DNA with the three qPCR kits tested. As the non-human sample was included to test the specificity of the qPCR assays, it will not be discussed further. Tables S1 and S2 include testing details and results.

### 3.2. NGS

#### 3.2.1. FORCE Capture

**Figure 3:**
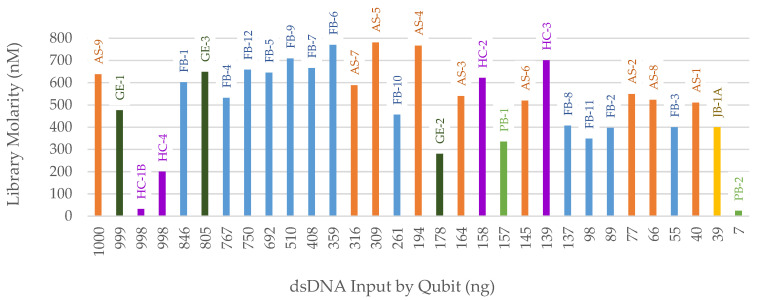
Library preparation dsDNA input determined by Qubit (ng) versus the resulting library molarity (nM) determined by the 2100 Bioanalyzer 170–1000 bp range. Sample names are listed above each bar. Color of bar indicates sample context: orange = Arch Street, dark green = WWII Germany, purple = Harewood Cemetery, blue = Basilica of St. Mary’s Assumption, light green = WWII Punchbowl, yellow = JB55.

**Figure 4:**
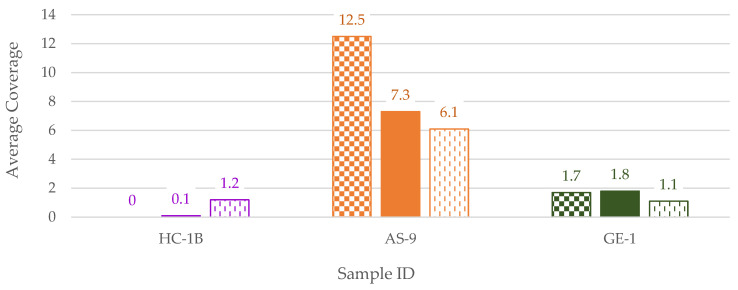
Average coverage of FORCE target regions for samples with reduced library DNA inputs. Results for each input are indicated by bars with the following patters: the maximum inputs (1 µg) = checkerboard, 500 ng inputs = solid, 200 ng inputs = vertical dashed lines. Color of bar indicates sample context: purple = Harewood Cemetery, orange = Arch Street, dark green = WWII Germany.

After library preparation, samples were run on the 2100 Bioanalyzer as a quality control check. The resulting library molarities were compared to the total dsDNA input (Figure 3). One outlier, HC-1B, was identified with nearly maximum total DNA input (998 ng) but no visible library product. This sample and two others with maximum DNA input were subsequently processed with reduced DNA input (500 ng and 200 ng) to test for inhibition or DNA overload. These reduced-input libraries were then captured with the FORCE panel and sequenced; the data were then compared with the 1 µg (maximum input) results. For HC-1B, the 200 ng input library generated the highest library molarity (570 nM compared to 115 nM for 500 ng input and 32 nM for 1 µg input in the 170–1000 bp range). Therefore, in the HC-1B extract, an inhibitor was likely present that, when diluted, resulted in successful library preparation and 1X coverage of target SNP regions (Figure 4). There was no indication of inhibition in HC-1B from any of the three qPCR kits, as the IPCs were within the expected ranges for all three. The typical forensically oriented qPCR and STR kits have buffers specifically designed to overcome inhibition common to forensic samples, such as humic acid, that can be co-extracted with DNA. However, other downstream assays with different chemistries, such as library preparation, may be negatively impacted by these common inhibitors. The other two samples included in the library input reduction test did not show indications of inhibition or overloaded library preparation (Figure 4). Sample AS-9 behaved as expected, with reduced inputs resulting in lower SNP coverage. The average coverage for sample GE-1 was consistently low for all inputs and could possibly be explained by minimal human DNA input and off-target mapping of non-human DNA.

Based on these findings, reduced volume input was used for HC-1B in all downstream tests. For the remaining 30 human sample extracts, the maximum input volume was utilized in all library preparation and PCR amplification events.

Data quality metrics from all samples are presented in Table S2. The mean mappable fragment size was sample dependent and ranged between 55 and 155 bp. The proportion of reads mapped to the human genome also varied, from <1% to 85%. Thus, these 31 samples exhibit poor DNA quality which is expected from ancient and historical remains. As stated previously, it is important to note that each of the 31 samples produced amplifiable human autosomal DNA, albeit in very low quantities, despite the small mappable fragment size.

**Figure 5:**
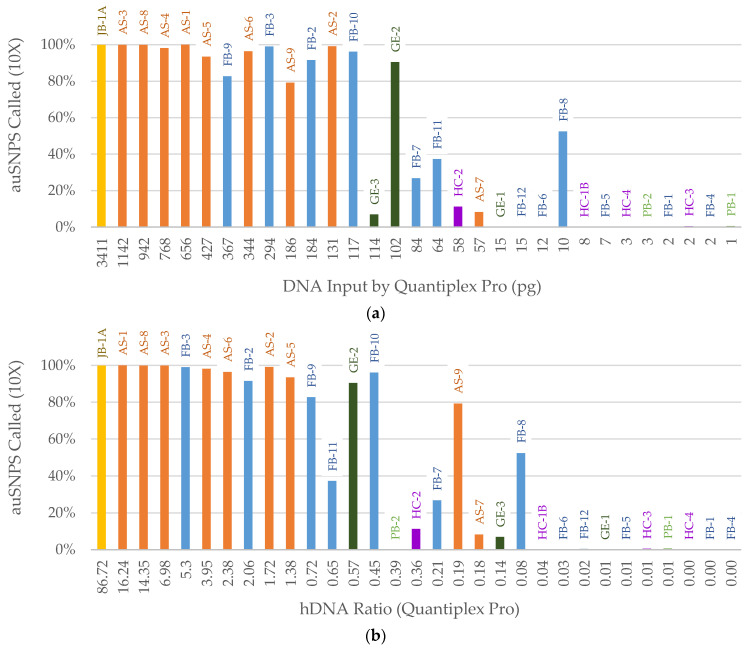
Percentage of autosomal SNPs (auSNPs) called at 10X based on (**a**) library preparation DNA input (pg) determined by the Quantiplex Pro small autosomal target (91 bp) and (**b**) human DNA (hDNA) Ratio (Quantiplex Pro small autosomal target). Sample names are listed above each bar. Color of bar indicates sample context: orange = Arch Street, dark green = WWII Germany, purple = Harewood Cemetery, blue = Basilica of St. Mary’s Assumption, light green = WWII Punchbowl, yellow = JB55.

SNP recovery was then assessed for all samples. To identify the best predictor of SNP recovery across both male and female samples, X- and Y-SNPs were ignored resulting in a maximum of 4342 autosomal SNPs (auSNPs) for analysis. The percentage of auSNPs called was plotted against the following: total dsDNA input from Qubit quantification, human DNA input based on the qPCR small autosomal target, and the hDNA ratio comparing the human DNA quantity (in pg) to total dsDNA quantity (in ng) as explained in the methods. Total dsDNA input was not a useful predictor, as these samples contain varying levels of exogenous DNA (Figure S6). The human DNA (qPCR-based) input (Figure 5a) was the best predictor of SNP recovery. Samples with approximately 100 pg or higher human DNA input typically resulted in ≥80% called SNPs, while samples below 100 pg generally resulted in <40% called SNPs (Figure 5a). In terms of the hDNA ratio (Figure 5b), samples ≥ 0.7 resulted in >80% called SNPs, and those with hDNA ratios of <0.05 failed. However, ratios between these two values were less predictive of SNP recovery.

Of note, two samples fell out of trend in terms of human DNA (qPCR-based) input. One sample with 114 pg input underperformed and a sample with 10 pg input overperformed. There is no clear explanation for the underperformance of the 114 pg input sample. Perhaps the root cause could be brought to light through replicate testing. The 10 pg sample had a mean paired distance of 80.8 bp, smaller than the small autosomal qPCR targets tested. Thus, the human DNA present in the 10 pg sample was likely too fragmented to be accurately quantified with the qPCR assays, yet sufficient fragmented human DNA was present to successfully sequence and cover the target SNPs.

**Figure 6:**
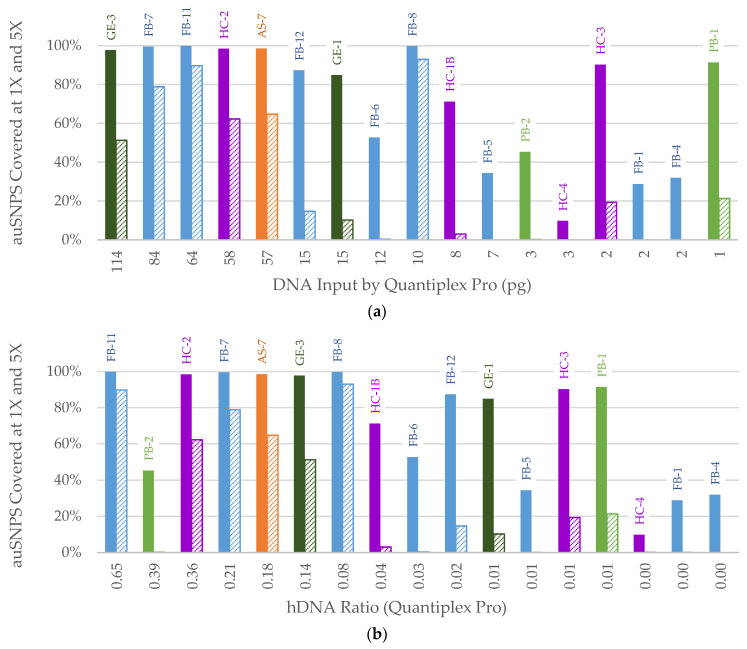
Percentage of autosomal SNPs (auSNPs) covered at 1X and 5X for samples with less than 75% of the auSNPs called at 10X based on (**a**) library preparation DNA input (pg) determined by the Quantiplex Pro small autosomal target (91 bp), 1X (solid bar) and 5X (diagonal lines) and (**b**) human DNA (hDNA) Ratio (Quantiplex Pro small autosomal target), 1X (solid bar) and 5X (diagonal lines). Sample names are listed above each bar. Color of bar indicates sample context: orange = Arch Street, dark green = WWII Germany, purple = Harewood Cemetery, blue = Basilica of St. Mary’s Assumption, light green = WWII Punchbowl, yellow = JB55.

SNP recovery at 1X and 5X was used in an exploratory capacity to assess samples with less than 75% of the auSNPs called at a 10X level. Since a larger SNP panel was not tested here, this was conducted to mirror the lower thresholds that can be used with probabilistic approaches for SNP analysis, such as a genotype likelihood approach [25]. For samples with at least 15 pg human DNA input (qPCR-based), >80% of SNPs were *covered* at a 1X threshold (Figure 6a). There were three samples that resulted in higher SNP recoveries than would have been expected at this threshold (Figure 6a, PB-1, HC-3, and FB-8). All three of these samples had total dsDNA inputs (Qubit-based) between 100 and 200 ng with mean paired distances between 60 and 80 bp. In these instances, the samples were fragmented beyond what could be detected with qPCR, but total dsDNA input was not as high as other samples that were found to contain high levels of exogenous DNA. For example, the other two samples with 2 ng input based on qPCR had total dsDNA inputs of 750–850 ng, indicating high levels of exogenous DNA. At human DNA inputs below 100 pg, samples may perform better than predicted by qPCR-based input alone if they have a relatively low exogenous DNA background and may be better characterized by considering both total dsDNA and human (qPCR-based) input. At the 5X threshold, aside from the 10 pg input outlier, samples with 57 pg and higher resulted in at least 40% of SNPs covered (Figure 6a). Conversely, samples with less than 57 pg input (all ≤ 15 pg) resulted in less than 20% of SNPs covered at the 5X threshold. There were no samples that had inputs between 57 pg and 15 pg in this dataset to evaluate 5X SNP recovery within this range. When assessing SNP recovery relative to the hDNA ratio (Figure 6b) there was a clearer trend than observed when considering only human DNA quantity, particularly for those samples discussed previously (Figure 6, PB-1, HC-3, and FB-8). Samples with an hDNA ratio ≥ 0.08 resulted in >40% SNP recovery at 5X and those < 0.08 resulted in <20% SNPs recovered. The exception was one sample with a ratio of 0.39 that produced only six SNPs covered at 5X. This sample had a very low DNA input (3 pg based on qPCR). Thus, the hDNA ratio may best be used as a measure of DNA quality for samples with 5–100 pg human DNA (qPCR-based) input.

It is worth noting that the FORCE panel is relatively small with only 4342 auSNPs (5402 total nuclear SNPs), and >1000 called SNPs at a 10X threshold with sufficient intra-locus balance are needed for accurate and statistically supported kinship comparisons between case and reference samples [26]. Those samples shown here with less than 20% of SNPs recovered (at a 10X calling threshold) would likely not result in sufficient SNPs for accurate predictions. As noted previously, samples with less than 100 pg of target input DNA may be better suited for whole genome enrichment and/or large SNP panels, such as the 95K panel [25], in order to obtain enough SNPs at 1X that they can be used for probabilistic kinship predictions.

No controls resulted in usable data and were free of any detectable contamination.

#### 3.2.2. Mitogenome Capture

**Figure 7:**
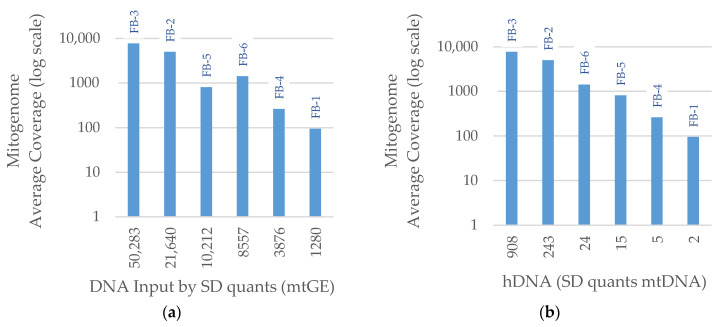
Average coverage of the mitogenome for a subset of Basilica samples by (**a**) library preparation DNA input (mtGE) determined by the SD quants small mitochondrial DNA (mtDNA) target (69 bp) and (**b**) human DNA (hDNA) ratio using the SD quants small mtDNA target. mtGE—mitogenome equivalents.

The results for the SD quants small mtDNA target were evaluated for a subset of Basilica samples with mitogenome hybridization capture data. The average coverage was plotted against mtDNA input based on the qPCR small mtDNA target (Figure 7a) and hDNA ratio (Figure 7b). For this analysis, the hDNA ratio was based on the qPCR small mtDNA target instead of the small autosomal target. The mtDNA input was positively correlated with the average coverage, but sample FB-5 fell slightly out of trend (Figure 7a). FB-5 had the highest mtDNA degradation index (69 bp mtDNA qPCR target/143 bp mtDNA qPCR target), indicating a greater degree of fragmentation than the other Basilica samples. FB-5 also had a greater amount of total dsDNA compared to FB-6, which suggests high exogenous DNA background. This was taken into account with the hDNA ratio (Figure 7b), and therefore, served as a slightly better predictor of average coverage of the mitogenome than mtDNA qPCR alone.

**Figure 8:**
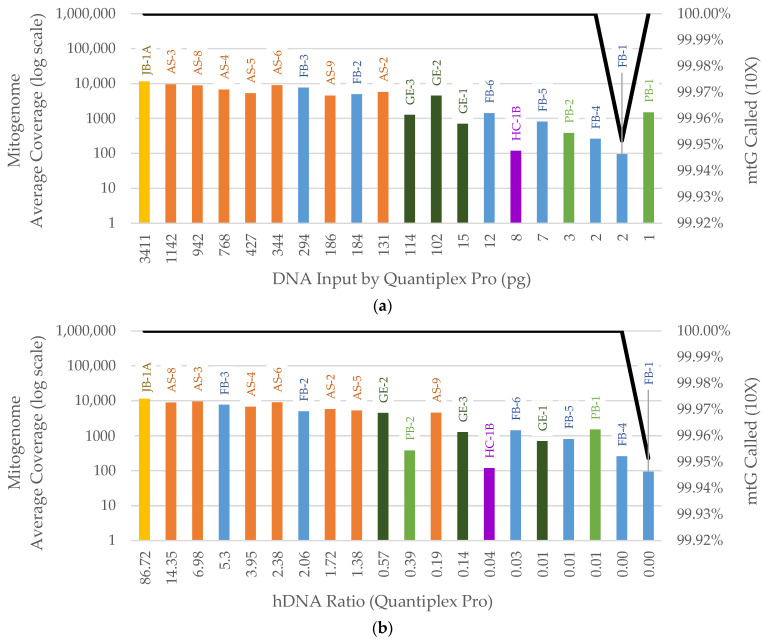
Average coverage of the mitogenome per sample by (**a**) library preparation DNA input (pg) determined by the Quantiplex Pro small autosomal target (91 bp), percentage of the mitogenome (mtG) called at 10X on the right *y*-axis (black line) and (**b**) human DNA (hDNA) ratio using the Quantiplex Pro small autosomal target, percentage of the mitogenome (mtG) called at 10X on the right *y*-axis (black line). Sample names are listed above each bar. Color of bar indicates sample context: orange = Arch Street, dark green = WWII Germany, purple = Harewood Cemetery, blue = Basilica of St. Mary’s Assumption, light green = WWII Punchbowl, yellow = JB55.

Many laboratories, including the AFMES-AFDIL, do not have access to a mtDNA quantification system. Therefore, mitogenome capture results were also assessed using total dsDNA from Qubit and nuclear DNA qPCR kits that are more widely implemented. The average coverage was plotted against total dsDNA input from Qubit, human DNA input based on the qPCR small human autosomal target, and the hDNA ratio based on the qPCR small autosomal target. This was conducted for the full bone sample dataset since all samples were quantified with autosomal qPCR. Consistent with the SNP data shown in Figure S6, total dsDNA from Qubit quantification (Figure S7) was not a suitable predictor of average coverage of the mitogenome, due to varying levels of exogenous DNA background. DNA input by qPCR (Figure 8a) and the autosomal hDNA ratio (Figure 8b) were both strong predictors of average mitogenome coverage. In Figure 8a, the majority of samples with ≥7 pg input resulted in average coverages above 500X; in Figure 8b, this was achieved for the majority of samples with hDNA ratios ≥0.01.

Figure 8 shows sample HC-1B having slightly lower average coverage than expected. This could be due to the sample inhibition identified in the library input reduction test. Sample PB-1 also fell out of trend in Figure 8a with an average coverage of 1501X. This sample’s average coverage was better explained by the hDNA ratio (Figure 8b). PB-1 was one of the two samples originating from the WWII Punchbowl context in which burials were subjected to formalin preservation. The chemical treatment of PB-1 may explain this sample’s unique signature: higher than the expected 1X auSNP coverage and higher mitogenome average coverage when compared to historical samples with similar human DNA input. It is notable that the other Punchbowl sample, PB-2, was better explained by human DNA input (Figure 8a) than the hDNA ratio (Figure 8b).

Across the entire dataset, the highest average coverage of the mitogenome observed was 11,515X, while the lowest was 95X—well above the 10X average coverage metric required by AFMES-AFDIL for data reporting. All samples except FB-1 generated full mitogenome coverage (16,569 bp) at a 10X threshold for the reported sequence range (Figure 8a). FB-1 had a 69 bp gap in the reported sequence range due to lower coverage (6–9X) observed in the polycytosine stretch in hypervariable region 1. Homopolymer regions often have lower coverage due to mapping difficulties [27], especially with very short fragments.

All samples were single sourced with no indication of a mixture. No controls resulted in usable data and were free of any detectable contamination.

### 3.3. STR Typing

**Figure 9:**
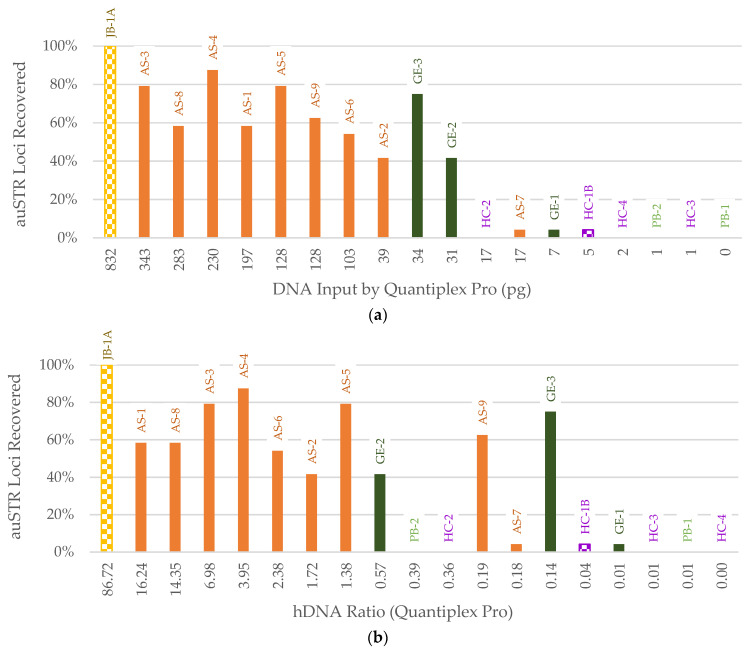
Percentage of autosomal STR (auSTR) loci in the PowerPlex Fusion kit recovered by (**a**) amplification DNA input (pg) determined by the Quantiplex Pro small autosomal target (91 bp) and (**b**) human DNA (hDNA) ratio using the Quantiplex Pro small autosomal target. All samples were amplified with the maximum volume input (15 µL), except JB-1A and HC-1B (checkerboard pattern) due to DNA extract volume limitations. Sample names are listed above each bar. Color of bar indicates sample context: orange = Arch Street, dark green = WWII Germany, purple = Harewood Cemetery, light green = WWII Punchbowl, yellow = JB55.

Nineteen samples underwent auSTR typing, of which 14 resulted in at least one reportable locus. The percentage of auSTR loci recovered was plotted against human DNA input from the qPCR small autosomal target and the hDNA ratio based on the qPCR small autosomal target. DNA input by qPCR (Figure 9a) was the best predictor of STR recovery, as samples with ≥30 pg input resulted in >40% recovery of auSTR loci. Below this threshold, a maximum of one locus was recovered. The hDNA ratio (Figure 9b) was less predictive, which suggests that the non-human DNA background did not impact the percentage of STR loci recovered. It is worth noting that the 550 RFU homozygote stochastic reporting threshold validated at the AFMES-AFDIL for PowerPlex Fusion data may have a more noticeable impact on auSTR locus reportability for degraded samples, particularly those with many homozygous loci.

**Figure 10:**
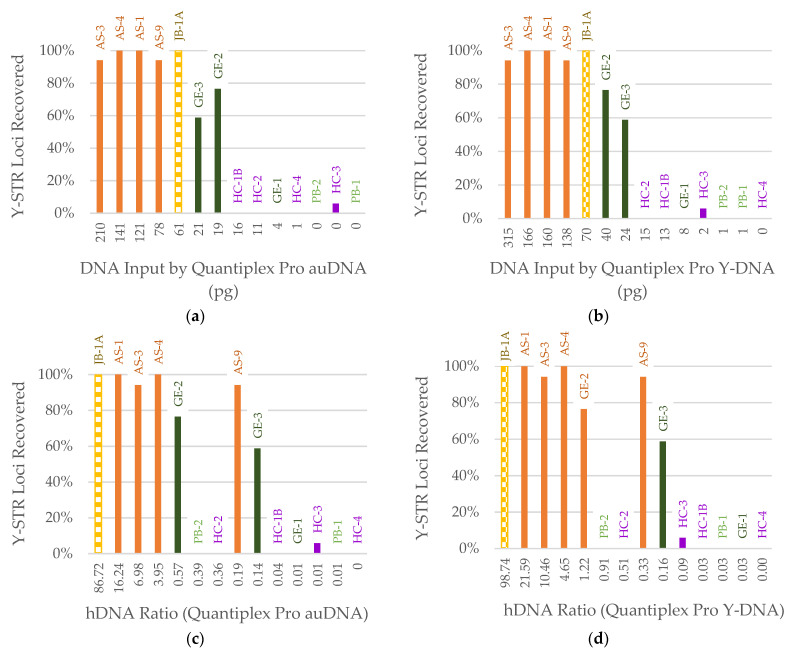
Percentage of Y-chromosomal STR (Y-STR) loci in YFiler recovered based on (**a**) input by Quantiplex Pro small autosomal target (91 bp), (**b**) input by Quantiplex Pro Y-DNA target (81 bp), (**c**) hDNA ratio (Quantiplex Pro small autosomal target), and (**d**) hDNA ratio (Quantiplex Pro Y-DNA target). All samples were amplified with 9.2 µL except JB-1A (checkerboard pattern) due to DNA extract volume limitations. Sample names are listed above each bar. Color of bar indicates sample context: orange = Arch Street, dark green = WWII Germany, purple = Harewood Cemetery, light green = WWII Punchbowl, yellow = JB55. Sample HC-4 is likely female based on previous conclusions drawn from the sample data for this historical context.

Of the 14 samples that were tested using the LCNY protocol, including one suspected female, eight samples resulted in at least one reportable Y-STR locus. The small autosomal and Y-DNA targets were both strong predictors of the percentage of Y-STR loci recovered (Figure 10a,b), but a clearer trend was observed in the prediction from Y-qPCR. The Y-DNA target was also smaller than the autosomal target, benefiting more degraded samples. Greater than 58% of the Y-STR loci were recovered in samples with ≥19 pg autosomal DNA (Figure 10a) and ≥24 pg Y-DNA (Figure 10b). Below these input values, only one sample had a single locus recovered. As expected for the suspected female sample, zero Y-STR loci were recovered. The hDNA ratio by the small autosomal target (Figure 10c) and Y-DNA target (Figure 10d) were poorly correlated with Y-STR recovery.

Additional LCNY amplifications were performed for sample HC-1B with reduced template volumes (1 µL and 5 µL) due to the inhibition observed during NGS processing. The 1 µL and 5 µL reduced template volumes resulted in Y-STR recoveries of 23.53% and 29.41%, respectively. As stated previously, the effects of inhibition were not observed in the IPCs for any of the qPCR assays. The LCNY protocol uses YFiler kit reagents, which is an older generation kit, and therefore, it may be less tolerant to inhibitors than current generation qPCR kits like those tested here.

No controls resulted in usable data and were free of any detectable contamination

## 4. Discussion

These results show that DNA quantification, both human-specific and total dsDNA content, can serve as a screening method for both high- and low-quality samples processed in ancient and forensic DNA laboratories. By testing a range of historical bone samples in terms of age and quality, the results from this study demonstrate that degraded and low template human DNA can be reliably quantified. Typical screening methods, such as WGS for aDNA laboratories, are costly and minimally effective for samples containing <1% endogenous DNA [8]. Information obtained from WGS screening such as relative endogenous DNA content and fragment length can also be estimated with a refined qPCR multiplex and total dsDNA quantification [28]. Maximizing the information that quantification systems offer allows the best downstream processing method to be selected. The quantification of extracts is a cost-effective and efficient approach since it precedes any downstream testing such as test amplification or library preparation and WGS. Additionally, many samples can be quantified simultaneously. This study consisted of samples with varying degrees of degradation. Samples were tested with several modalities of forensic DNA profiling (SNP genotyping, STR typing, and mtDNA sequencing) to comprehensively highlight the ways in which the qPCR system implemented can predict sample success.

While this study highlights the potential benefits of qPCR screening, implementation in a forensic laboratory would require a number of studies to meet accreditation standards. These studies include an assessment of accuracy and precision with technical replicates, representative non-human samples for species specificity, mixtures, sensitivity, and case-type non-probative samples. Case samples vary between laboratories; therefore, ensuring the qPCR kit used is tailored to the samples and downstream processing can result in the most reliable prediction of success. Selecting a qPCR autosomal target of a similar size to the expected fragment length of sequenceable and informative fragments would best forecast success. For highly fragmented samples that are intended for NGS, a 60 bp qPCR target may better predict NGS success using a hybridization capture approach, while a 90 bp or larger target may be more appropriate for amplicon-based library preparation (or STR amplification). Where possible, having multiple targets of varying size would allow fragment size distributions to be inferred, which would help predict success as well as determine the number of cycles needed for sequencing (as longer DNA fragments require more cycles to sequence entire reads).

Similarly, the qPCR kit will be more predictive if the qPCR buffer’s robustness to inhibition is consistent with the chemistry utilized in downstream processing methods to best alert the user when reduced input or dilution may be a benefit. For example, it is possible that current generation Y-STR kits, such as YFiler Plus (Thermo Fisher Scientific) and PowerPlex Y23 (Promega), have similar inhibitor tolerance to the qPCR assays tested in this study. Therefore, the newer Y-STR kits might also produce results in the presence of inhibitors that are more consistent with expectations based on current generation qPCR results. These considerations are true of traditional forensic STR typing and could also be applied to other processing streams such as NGS. Selecting a qPCR target specific to the data being generated is also beneficial in predicting sequencing results. For a subset of Basilica samples, the hDNA ratio determined by the small mtDNA qPCR target served as a better predictor of mtDNA capture results, versus the hDNA ratio or simply the quantification results of the small autosomal target. Likewise, the small Y-DNA target was a better predictor of LCNY success, as it was a more direct measurement of the target. Depending on the amount of exogenous DNA present in the samples being studied, the quantification results alone may serve as a sufficient predictor of on-target coverage.

The DNA extraction method used can also affect fragment size distribution and thus the ideal qPCR target size. Extractions such as the Dabney method [1] that prioritize small fragment retention may benefit from a smaller target compared to extracts created with other protocols. For example, a small subset of samples extracted with both the Dabney method and another common total demineralization extraction method [29], and processed through the FORCE hybridization capture method, showed that the average fragment length of DNA mapping to GRCh38 after FORCE SNP capture was approximately 40 bp smaller with the Dabney method (~110 bp vs. ~70 bp) (Table S3). While a direct comparison of DNA extraction methods was not an aim of this study, future work could include such a comparison of commonly utilized forensic and ancient DNA procedures [16]. Future research could determine what impact any one DNA extraction method could have on the resulting qPCR and/or hDNA results, as well as downstream DNA profiling success.

In conclusion, the three commercially available qPCR kits tested in this study were highly comparable. All three qPCR assays were appropriate for reliable quantification of historical DNA samples exhibiting varying levels of degradation. In scenarios where both nuclear DNA and mtDNA could be targets of interest, currently available qPCR kits with small autosomal targets were not only useful for predicting auDNA results but were also sufficient for predicting mtDNA success in most cases. Overall, qPCR quantification alone was the best predictor of sample success for both NGS and STR processing workflows. The hDNA ratio was used secondarily to predict SNP success where human DNA input was low (<100 pg) and in mitogenome capture. The qPCR-based DNA input requirements identified in this study can guide laboratories toward the downstream processing method(s) with the highest potential for success depending on the information required for a particular case. Toward this end of achieving a particular human DNA input target for predicted NGS or STR success, DNA extracts can be combined and/or concentrated to increase target DNA. Sample extracts with high exogenous DNA content that may exceed NGS library input recommendations can have libraries combined and/or concentrated for input into a single hybridization capture reaction for improved recovery. Alternatively, low quantity sample extracts can also benefit from concentrating sample library to increase input into SNP capture. If human DNA input is unavoidably low, mitogenome capture may be the best choice for obtaining forensically relevant genetic information from an ancient or historical case sample. Ultimately, these data may help to set a benchmark for bone sample success.

## Figures and Tables

**Table 1:** Sample context descriptions for the thirty burials tested.

Context/Region	Number of Burials	Approximate Years Postmortem
JB55, Griswold, Connecticut, USA	2	200
Arch Street, Philadelphia, Pennsylvania, USA	9	275
World War II-Germany	3	80
World War II-Punchbowl ^1^, Honolulu, Hawaii, USA	2	80
Harewood Cemetery, Charles Town, West Virginia, USA	3	200
Basilica of St. Mary’s Assumption, Fürstenfeldbruck, Germany	12	700–800

^1^ Punchbowl-National Memorial Cemetery of the Pacific.

**Table 2:** Autosomal, Y-chromosomal, and internal positive control (IPC) target sizes in quantitative real-time PCR kits used to quantify human nuclear DNA.

Kit	Small Autosomal Target Size	Large Autosomal Target Size	Y-Chromosomal Target Size(s)	IPC Target Size
PowerQuant System	84 bp	294 bp	81 bp, 136 bp	435 bp
Investigator Quantiplex Pro	91 bp	353 bp	81 bp	434 bp
Quantifiler Trio	80 bp	214 bp	75 bp	130 bp

## Data Availability

Data are stored at the AFMES-AFDIL and may be made available to approved laboratories upon written request.

## Supplementary Materials

The following supporting information can be downloaded at: https://www.mdpi.com/article/10.3390/genes14050994/s1,. Table S1: Sample and lab processing information for each sample. Table S2: Detailed data quality metrics per sample. Figure S1: Small autosomal target results for Quantifiler Trio (80 bp) compared to PowerQuant (84 bp). Figure S2: Approximate years postmortem compared to the Quantiplex Pro small autosomal target for each human sample. An average of 750 years postmortem was utilized for the Basilica context (ranged 700-800 years). Color of data point indicates sample context: orange = Arch Street, dark green = WWII Germany, purple = Harewood Cemetery, blue = Basilica of St. Mary’s Assumption, light green = WWII Punchbowl, yellow = JB55. Figure S3: Small autosomal target results for Quantiplex Pro (91 bp) compared to SD quants system autosomal target (70 bp) for all samples tested with both kits. FigureS4: Y target results for Quantiplex Pro (81 bp) compared to Quantifiler Trio (black—75 bp target) and Promega PowerQuant (red- 81 bp target). Figure S5: Small Y target results for Quantifiler Trio (75 bp) compared to Promega PowerQuant (81 bp). Figure S6: Percentage of autosomal SNPs (auSNPs) called at 10X per sample by library preparation dsDNA input determined by Qubit (ng). Sample names are listed above each bar. Color of bar indicates sample context: orange = Arch Street, dark green = WWII Germany, purple = Harewood Cemetery, light green = WWII Punchbowl, yellow = JB55. Figure S7: Average coverage of the mitogenome per sample by library preparation dsDNA input determined by Qubit (ng). Sample names are listed above each bar. Color of bar indicates sample context: orange = Arch Street, dark green = WWII Germany, purple = Harewood Cemetery, light green = WWII Punchbowl, yellow = JB55. Table S3: Comparison of average fragment lengths after trimming for a set of Dabney and demineralization extracts
